# Long-term humoral and cellular immune responses following Covaxin vaccination: a 2-year prospective longitudinal study

**DOI:** 10.3389/fimmu.2026.1754692

**Published:** 2026-03-04

**Authors:** Archana Tripathy, Sreeparna Podder, Swatishree Sradhanjali, Mamuni Swain, Debdutta Bhattacharya, Debaprasad Parai, Sanghamitra Pati, Sunil K. Raghav

**Affiliations:** 1 Immunogenomics and Systems Biology Laboratory, Institute of Life Sciences (ILS), Bhubaneswar, Odisha, India; 2 Immunogenicity Assay Platform (NABL Laboratory), Institute of Life Sciences (ILS), Bhubaneswar, Odisha, India; 3 Department of Microbiology and One Health, ICMR-Regional Medical Research Centre, Bhubaneswar, India

**Keywords:** AIM (activation induced markers), Covaxin, COVID-19, longitudinal cohort, neutralizing antibody (NAb), receptor binding domain (RBD), SARS-CoV-2, spike (S)

## Abstract

**Background:**

Globally, multiple SARS-CoV-2 vaccines received emergency authorization, primarily based on adenoviral vector, mRNA, or inactivated virus platforms. Among them, Covaxin, an inactivated vaccine, was widely used in India and several Southeast Asian countries. Due to their emergency rollout in 2021, the long-term immunogenicity data to assess the impact of these vaccinations have been limited. This study investigated the prolonged immune responses induced by Covaxin, an inactivated virus-based COVID-19 vaccine, in 250 individuals monitored for 2 years.

**Methods:**

This longitudinal study (January 2021–January 2023) tracked 250 participants, collecting blood at seven time points. We measured SARS-CoV-2 spike RBD IgG and neutralizing antibodies using ECLIA and surrogate virus neutralization tests, respectively. We also assessed cellular immunity in a subset of Covaxin recipients through flow cytometry of spike protein-stimulated lymphocytes.

**Results:**

Anti-RBD IgG levels declined rapidly post-vaccination. A significant rise was observed following Omicron infection, with sustained high antibody titers and high virus neutralization capacity. Covaxin recipients demonstrated high CD4^+^ T-cell activity during the Omicron wave, correlating with mild or asymptomatic infections. These findings suggest that Omicron exposure may have served as a natural booster and hold potential for next-generation vaccine development for COVID. Enhanced T-cell responses, particularly after the third dose, further underscored the vaccine’s ability to maintain cellular immunity. Compared with Covishield, Covaxin elicited milder immune responses, possibly contributing to its favorable safety profile.

**Conclusions:**

Overall, this study provides one of the first longitudinal analyses of the humoral and T-cell responses to Covaxin, a vaccine widely administered in India and neighboring Southeast Asian countries.

## Introduction

The coronavirus disease 2019 (COVID-19) outbreak, which was first reported in Wuhan, China, in late December 2019, has affected millions of people and caused significant deaths worldwide (1). COVID-19 is caused by the highly contagious severe acute respiratory syndrome coronavirus 2 (SARS-CoV-2), an enveloped RNA virus that belongs to the Coronaviridae family (2). The World Health Organization (WHO) declared the novel coronavirus outbreak a global pandemic in March 2020 due to its high transmission efficiency. The virus is transmitted through the respiratory aerosols and direct or indirect contact with the infectious particles/droplets. Infections with SARS-CoV-2 cause mild to severe respiratory illness, characterized by dry cough, cold, runny nose, sore throat, fever or chills, chest congestion, shortness of breath, or even pneumonia in more severe cases (3).

The rapid genetic evolution and emergence of new variants of concern (VOCs) of SARS-CoV-2, such as B.1.1.7 (Alpha), B.1.351 (Beta), P1 (Gamma), B.1.617.2 (Delta), and B.1.1.529 (Omicron), posed a considerable challenge in controlling the spread of COVID-19 due to their high transmissibility, virulence, immune evading/escaping trait, and their diminished response to vaccines and therapeutics (4, 5). Several vaccines against SARS-CoV-2 have been licensed or are in the process of development around the world over the past 2–3 years (5–7). There are currently four types of vaccines available against the SARS-CoV-2 virus with different mechanisms of action, namely, mRNA, adenovirus vector-based, COVID-19 protein subunit, and inactivated virus particle-based vaccines. Several vaccine efficacy studies have shown promising results ranging from 50% to 96% effectiveness against symptomatic SARS-CoV-2-infected individuals, including vaccines such as Covaxin, ChAdOx1 nCoV-19, and mRNA vaccines (8–11). Covaxin (BBV152) is an inactivated whole-virion SARS-CoV-2 vaccine based on the ancestral (D614G) strain of the virus. It was first licensed for emergency use in India in January 2021 and was primarily administered to adults, including healthcare workers, frontline workers, and the elderly population. Phase 3 clinical trials of Covaxin reported an overall efficacy of 77.8% against symptomatic COVID-19, and real-world studies in India demonstrated similar effectiveness, including protection against the Delta variant (12, 13). However, a rapid decline in vaccine efficacy from 1 to 6 months has been noticed in fully vaccinated individuals. Furthermore, the rapid emergence and spread of SARS-CoV-2 VOCs compromise the efficacy of vaccines due to their enhanced ability to evade the immune response. Rare serious adverse events have been reported after COVID-19 vaccination, including myocarditis/pericarditis following mRNA vaccines and thrombosis with thrombocytopenia following adenoviral-vector vaccines, whereas Covaxin continues to demonstrate a strong safety record in real-world use (12, 13). Consequently, a detailed insight into the underlying molecular mechanism of SARS-CoV-2 infection and host immune response is of paramount importance for efficient vaccine development, taking into account the safety of the vaccine. Several longitudinal studies of humoral antibody response induced by COVID-19 vaccines have been reported in the recent past (14–16). However, we are the first to show the longitudinal IgG antibody profiles and neutralizing antibody titers (Nab) in the serum of Covaxin (BBV152)-vaccinated individuals, the vaccine which was predominantly administered in India and neighboring Southeast Asian countries. In the present prospective cohort study, we aimed to characterize the longitudinal humoral and cellular immune responses in Covaxin-vaccinated and/or SARS-CoV-2 convalescent individuals. We assessed IgG responses against the receptor-binding domain (RBD) of the Alpha variant, evaluated long-term serum neutralizing activity against the ancestral strain, and analyzed CD4^+^ and CD8^+^ T-cell responses, including their cross-reactivity with Delta and Omicron variants. The observations from our results suggested that the Omicron variant of SARS-CoV-2 could be a better vaccine candidate for future innovations in this aspect.

## Materials and methods

### Study design and patient cohort

**Figure 1:**
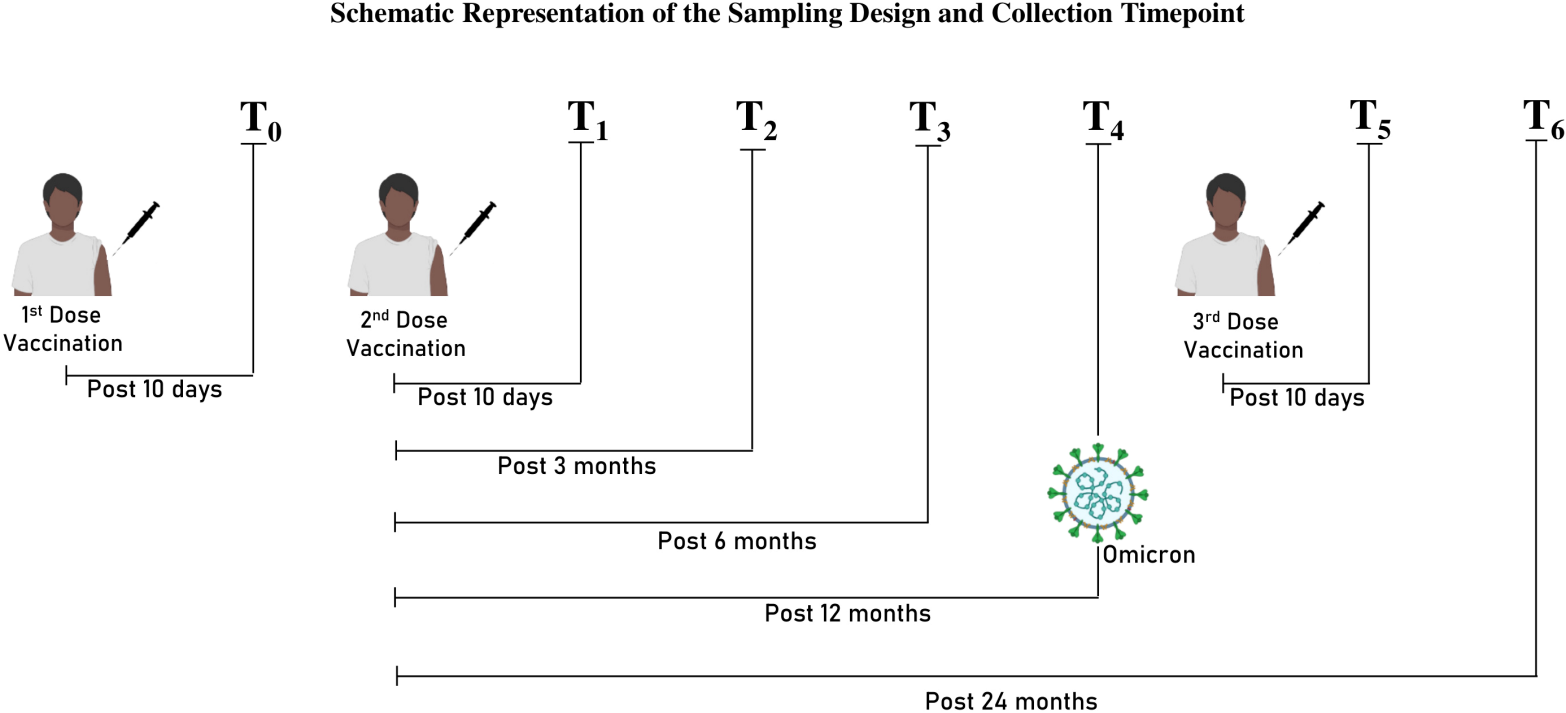
Schematic representation of the sampling design and collection time point.

The longitudinal cohort samples were collected from January 2021 to January 2023 with 250 participants at the Institute of Life Sciences, Bhubaneswar, to investigate the RBD IgG antibody titer, pseudo-virus neutralization serum titers, and T-cell responses against SARS-CoV-2 at seven and five distinct time points, respectively (before the second dose: T_0_; after 10 days of the second dose: T_1_; after 3 months of the second dose: T_2_; after 6 months of the second dose: T_3_; after 12 months of the second dose: T_4_; after 10 days of the third dose: T_5_; and after 24 months of the second dose: T_6_) (**Figure 1**). We managed to follow 34 enrolled participants for all the specified time points of the study. All participants were interviewed, and their demographic information, SARS-CoV-2 infection history, and vaccination details were recorded (**Table 1**). The study was reviewed and approved by the Institutional Ethics Committee (BT/CS0053/05/21).

**Table 1:** Participant characteristics.

Parameters	Participant
Age, years, median	32
Sex, female/male, (*n)*	120/130
Sample Collection Duration	January, 2021- January 2023
Blood Collection time points *(n)*	6
Vaccination status with 3 doses, (*n)*	250
Follow up individuals n, Convalescent/Uninfected (*n)*	43 19/25
Infection history post vaccination Cold (*n)* Fever (*n)*	12 32

### Isolation, preservation of PBMCs, and serum collection

Human peripheral blood was collected in EDTA-coated and spray-coated silica vacutainers (BD) for peripheral blood mononuclear cell (PBMC) isolation. PBMCs were separated using a Ficoll-Hypaque density gradient (Lymphoprep, StemCell Technologies, Vancouver, British Columbia, Canada) according to the manufacturer’s instructions and cryopreserved in liquid nitrogen under recommended conditions until further use. Blood was collected in clot-activator vacutainer tubes (BD) under sterile conditions for serum separation. After clotting, the blood was centrifuged, and the serum was aliquoted and stored at −80°C until further use.

### Chemiluminescent microparticle immunoassay

Serum samples were used to evaluate IgG antibody titers against the RBD region of SARS-CoV-2 spike antigen using the ARCHITECT i1000SR (Abbott Diagnostics, USA) platform, which is based on chemiluminescent microparticle immunoassay (CMIA). The antibody titer was estimated using the ARCH SARS-CoV-2 IgG II Quant (Abbott Diagnostics, USA) kit as per the manufacturer’s instructions. The cutoff value for this quantitative kit was ≥50 AU/mL.

### Surrogate virus neutralization assay

Serum samples of vaccinated individuals were tested for neutralizing antibodies using the SARS-CoV-2 Surrogate Virus Neutralization Test Kit (GeneScript, Piscataway, New Jersey, United States), according to the manufacturer’s protocol. One hundred microliters of the sample mixture was then added to the pre-coated hACE2 plate and incubated for 15 min at 37°followed by four washing steps with 1× washing buffer. After complete removal of the residual wash buffer, 100 µL of TMB solution was added to each well, and plates were incubated in the dark for 15 min at RT. Finally, 50 µL of stop solution was added to each well, and absorbance was measured immediately at 450 nm using a Multiskan reader (Thermo Scientific, Waltham, Massachusetts, United States). The formula for calculating the percent inhibition/neutralization was = (1 − OD value of sample/OD value of negative control) × 100%.

### Meso-scale discovery assay

Serum cytokines were measured using the Human U-PLEX Proinflam Combo 1 Kit from MSD (Gaithersburg, MD). This kit was designed to measure a range of cytokines, including IFN-γ, IL-1β, IL-2, IL-4, IL-6, IL-8, IL-10, IL-12p70, IL-13, and TNF-α, with a broader dynamic range and higher sensitivity. MSD plates were analyzed on the MESO QuickPlex SQ 120 MM (MSD) platform. The assay was executed following the manufacturer’s guidelines, and all standards were run in duplicate. Recovery of cytokines from serum was evaluated as the ability to detect known concentrations of recombinant cytokine standards run in the same plate provided with the kit. This assessment was conducted using the MSD Discovery Workbench software (version LSR_4_0_13).

### PBMC stimulation *in vitro*

Cryopreserved PBMCs were quickly thawed at 37 °C and resuspended in RPMI media (Gibco, Waltham, Massachusetts, United States) supplemented with 1% penicillin/streptomycin solution (Gibco, Waltham, Massachusetts, United States) and 5% heat-inactivated human AB serum (Sigma-Aldrich, St. Louis, Missouri, United States). After washing, cells were cultured at 1.5 × 10^6^ cells per well for 12 h in 96-well U-bottom plates. Next, PBMCs were stimulated with peptide pools of SARS-CoV-2 wild type, Delta, and Omicron (Milteyni Biotec, Bergisch Gladbach, Germany) at 1 µg/mL for 24 h. Positive controls consisted of cells treated with 1 µg/mL of Cytostim (Milteyni Biotec) with the same conditions (**Supplementary Figure 1**), and unstimulated cells were used as negative controls. After 24 h of stimulation, cells were washed with 1× PBS and then stained with antibodies for FACS analysis.

### Flow cytometry

The expression of activation-induced markers (AIM) by stimulated PBMCs was analyzed using a full-spectra flow cytometer (Cytek Aurora). Briefly, the cells were resuspended in 100 µL of FACS buffer (3% FBS in PBS) and stained for viability (Zombie UV, BioLegend, San Diego, California, United States) as well as surface markers for 30 min in the dark at 4 °C with the following antibodies: anti-CD3-BV570 (BioLegend, San Diego, California, United States), anti-CD8-cFluor™ B532, anti-CD4-cFluor™ YG584, anti-CD45RA-cFluor™ V450 (Cytek, Fremont, California, United States), and anti-CCR7-BV421 (BioLegend, San Diego, California, United States). For intracellular staining, cells were washed with FACS buffer and subsequently fixed with BD Cytofix (BD Biosciences, Franklin Lakes, New Jersey, United States) for 20 min at 4 °C. After washing with BD Cytoperm (BD Biosciences, Franklin Lakes, New Jersey, United States), cells were labeled with anti-CD137-BUV615 and anti-CD154-BUV737 (BD Biosciences, Franklin Lakes, New Jersey, United States). The expression levels were measured, and data were analyzed using OMIQ.

### Data analysis

Statistical analyses were performed using GraphPad Prism 9 software. Significant differences were tested by Student’s *t*-test and one-way ANOVA. The data were expressed as the mean ± SEM. A two-tailed *p*-value of <0.05 was considered statistically significant. The Loess regression method was used to conduct the correlation assessment.

Spike‐specific response AIM was background‐subtracted by the values obtained with the unstimulated group. The percentage of AIM+ cells after stimulation with peptide pools was divided by the percentage of AIM+ cells generated from the unstimulated group to calculate the stimulation index (SI). The SI <1 was represented as 1.

## Results

### Longitudinal RBD-specific IgG profiles in Covaxin (BBV152)-vaccinated subjects

**Figure 2:**
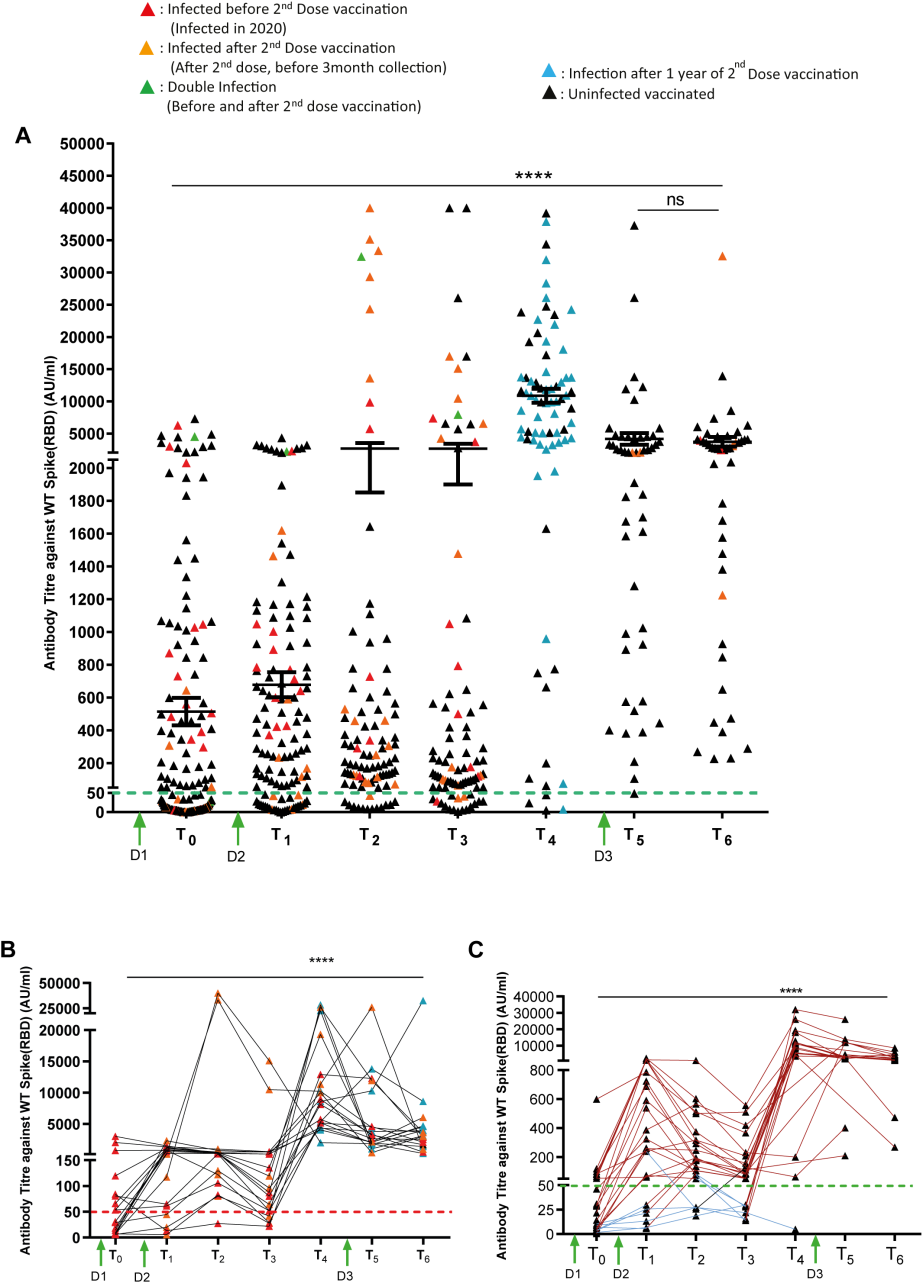
Longitudinal profiling of RBD-specific IgG antibody titers in Covaxin-administered subjects. **(A)** Covaxin-induced IgG titers in the vaccinated subjects at seven follow-up time points, as estimated using the CLIA instrument (*n* = 250). RBD-specific IgG antibody titers over the course of the longitudinal follow-up trial in **(B)** convalescent vaccinated and **(C)** uninfected subjects (convalescent, *n* = 19; uninfected, *n* = 25). In the graph, each individual is represented by a point, and dotted lines indicate the cutoff value to define the subjects with positive serum antibody titers. The subjects below the cutoff line represent the seronegative samples. Bars represent mean ± SEM. ****p < 0.0001. Significant differences were tested by unpaired Student’s *t*-test and one-way ANOVA with Tukey’s multiple comparison test.

To understand the changes in RBD-specific IgG antibody titers after vaccination, we established a cohort of 250 subjects who were vaccinated with Covaxin (BBV152). The antibody titers were estimated at regular 3-month intervals after the first (D1) and second (D2) dose administration. The serum samples were subjected to CLIA-based IgG antibody estimation against wild-type (WT) SARS-CoV-2 spike protein, and its persistence at seven longitudinal time points was analyzed: T_0_, 28 days after the first dose (*n* = 184); T_1_, 10 days after the second dose (*n* = 123); T_2_, 3 months after the second dose (*n* = 92); T_3_, 6 months after the second dose (*n* = 87); T_4_, 12 months after the second dose (*n* = 76); T_5_, 18 months after the second dose and 10 days after the third dose (*n* = 51); and T_6_, 24 months after the second dose and 6 months after the third dose (*n* = 46). We also segregated the subjects based on their infection status before and after vaccination to assess how infection along with vaccination impacted the IgG titers. We found that irrespective of the time points till T_3_, 15%–20% of subjects were non-responders with no antigen-specific antibody titers after vaccination (**Table 2**). At T_0_, more than 50% subjects were non-responders, and the responders also had lower IgG titers than after the second dose administration, i.e., T_1_ (**Table 2**). The IgG titers were found to be significantly decreased gradually after T_1_ till T_4_, until the time of the Omicron variant infection wave of SARS-CoV-2 (**Figure 2**). Interestingly, we found that the majority of the subjects at T_4_, i.e., during the Omicron wave of infection, had very high IgG titers, and there were no or only a few non-responders (**Figures 2A–C**). The results indicated that the Omicron variant has a much wider range of antigenic epitopes on the spike protein, leading to the generation of higher IgG antibody titers, which is shown by the higher mutations found in the Omicron spike RNA sequence as compared to WT SARS-CoV-2 or the Delta variant sequences (**Supplementary Figure 2**).

**Table 2:** Descriptive analysis of IgG-RBD antibody titre and neutralisation antibody titre of Covaxin (BBV152) vaccinated individuals.

Antibody	T0	T1	T2	T3	T4	T5	T6	*p*-Value
Anti RBD IgG antibody	N= 184	N= 123	N= 92	N= 87	N= 76	N= 51	N= 46	****, One-way ANOVA, T4 compared to all other time points.
Median (IQR) <44.3 44.3<Ab<386.3 386.3<Ab<2076.3 >2076.3	23.2 (5.2-462.1) 107 (58.1%) 26 (14.1%) 38 (20.6%) 13 (7%)	386.3 (58.6-931.8) 27 (21.9%) 35 (28.4%) 50 (40.6%) 11 (8.9%)	219.8 (106.4-528.1) 12 (13%) 49 (53.2%) 22 (23.9%) 9 (9.7%)	158.7 (59.4-593.3) 15 (17.2%) 43 (49.4%) 13 (14.9%) 16 (18.3%)	9902.7 (3886.5-13734.8) 3 (3.9%) 5 (6.5%) 7 (9.2%) 61 (80.2%)	2111.8 (1007.2-4462.15) 0 (0%) 4 (7.8%) 20 (39.2%) 27 (52.9%)	2759 (1405.1-4080) 0 (0%) 4 (8.6%) 14 (30.4%) 28 (60.8%)	
Neutralization antibody	N= 34	N= 34	N= 34	N= 34	N= 34	N= 34	N= 34	*, Student’s t-Test
Median (IQR) <54.3 54.3 <Ab<72.5 72.5<Ab<86.8 >86.8	69.1 (61.3-76.1) 9 (26.5%) 8 (23.5%) 7 (20.6%) 10 (29.4%)	88.04 (60.3-93.8) 9 (26.5%) 8 (23.5%) 8 (23.5%) 9 (26.5%)	45.9 (16.0-72.4) 8 (23.5%) 9 (26.5%) 7 (20.6%) 10 (29.4%)	40.7 (30.1-60.3) 9 (26.5%) 8 (23.5%) 8 (23.5%) 9 (26.5%)	94.8 (93.0-94.9) 9 (26.5%) 6 (17.6%) 6 (17.6%) 13 (38.2%)	80.2 (67.9-86.0) 8 (23.5%) 9 (26.5%) 8 (23.5%) 9(26.5%)	74.9 (70.8-79.0) 9 (26.5%) 7 (20.6%) 9 (26.5%) 9 (26.5%)	

*p < 0.05 and ****p < 0.0001.

The third dose of the vaccine was administered in June 2022 (T4), and we found that, even after the third dose of vaccination, the IgG titers were significantly lower at T_5_ than at T_4_, the levels after Omicron (**Figure 2**). Similar levels of IgG titers were maintained till T_6_. These profiles strongly suggested that the Omicron variant has a higher potential of generating sustained IgG antibody titers than the SARS-CoV-2 Alpha variant originally used for making the vaccine BBV152. Moreover, we separately looked into the IgG titer longitudinal profiles in uninfected (*n* = 25) and convalescent individuals (*n* = 19) based on prior infection status, and we found that infected individuals showed higher IgG titers upon Covaxin administration, and the antibody titers were sustained for a longer time as compared to uninfected vaccinated subjects. Interestingly, both these groups behave similarly with the highest antibody titers at T_4_, which corresponded to the Omicron infection wave (**Figures 2B, C**).

### Pseudo-virus neutralization assay to assess the virus-neutralizing activity of serum from Covaxin vaccinees

**Figure 3:**
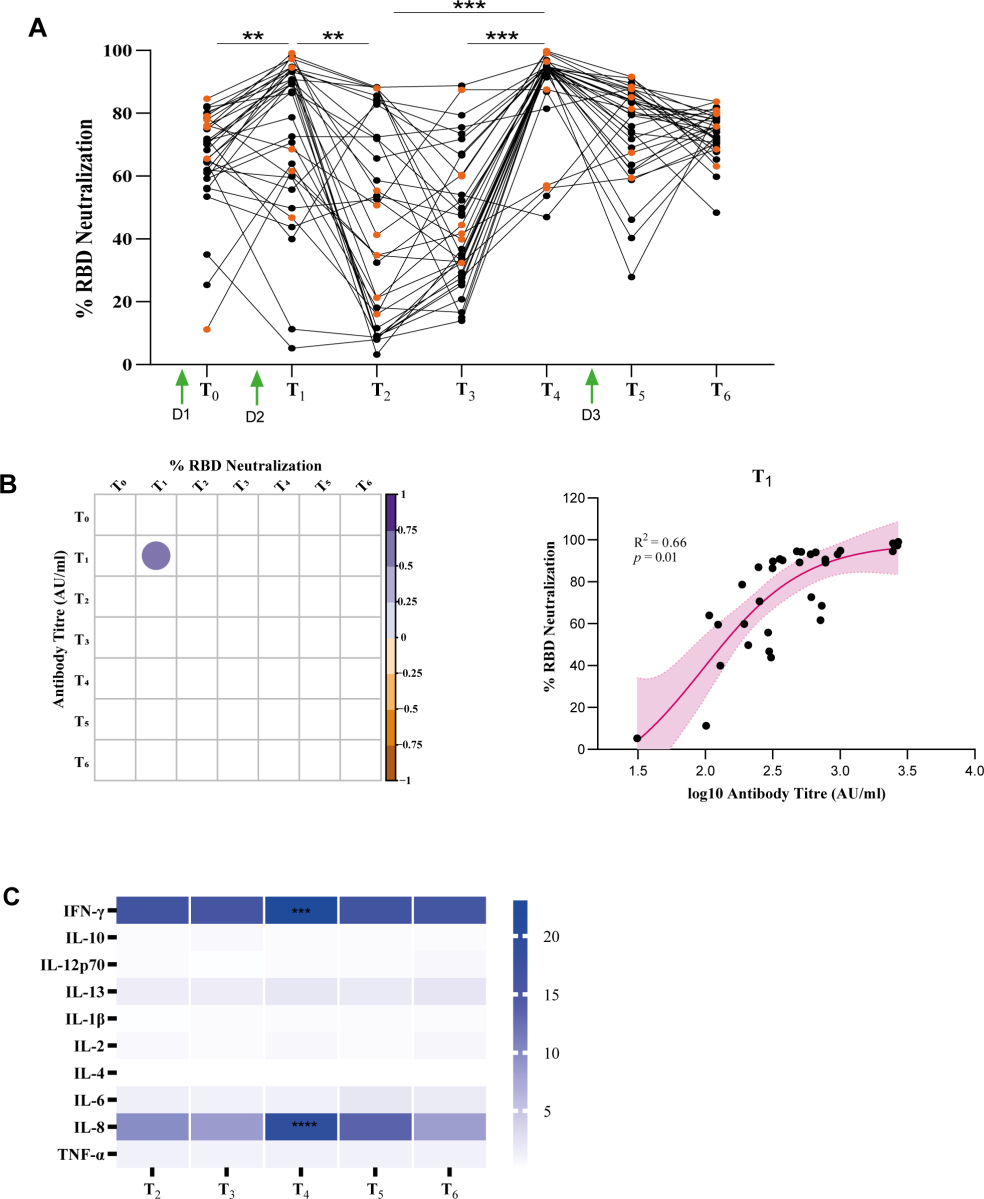
SARS-CoV-2 neutralization assay and serum cytokine profile in a long-term follow-up of a Covaxin-administered cohort. **(A)** Inhibition of SARS-CoV RBD–hACE2 interaction by sera from 34 follow-up individuals (convalescent, *n* = 9; uninfected, *n* = 25) throughout all time points. **(B)** (Left panel) Correlation between antibody titer vs. % of RBD neutralization by serum of vaccinated individuals at all time points. (Right panel) Loess regression analysis depicts significance in the T_1_ time point (after 10 days of dose 2). **(C)** Heatmap depicting the average expression of each cytokine in the serum samples of vaccinated individuals. Significant differences were tested by an unpaired Student’s *t*-test. **p < 0.01, ***p < 0.001, ****p < 0.0001.

It is crucial to verify whether higher antibody titers are associated with virus neutralization. To this end, we conducted a spike RBD-ACE2 interaction-based surrogate virus neutralization assay on a long-term follow-up cohort of vaccinated individuals (*n* = 34). Our findings revealed that during the Omicron wave phase (T_4_), the neutralization percentage was significantly elevated, with more than 50% of individuals showing values that fall within the interquartile range (IQR) of 72.5–86.8 (**Figures 3A**, **Table 2**). This finding is consistent with the observed antibody titers (**Figure 2**). The presence of Omicron facilitated an increased level of neutralizing antibodies against WT-RBD, raising questions about variant specificity.

Post-second dose (T_1_) vaccination showed greater inhibition capacity compared to T_2_ and T_3_, likely due to Covaxin being developed using the WT SARS-CoV-2 strain. Furthermore, a correlation study between antibody titers and percentage of inhibition in vaccinated subjects (**Figure 3B**) demonstrated a significant correlation of 0.66 at T_1_.

A set of 10 cytokines was assessed in serum samples obtained from individuals who had received the Covaxin vaccine. Among the findings, it was observed that two cytokines, namely, IFN-γ and IL-8, displayed a significant increase at T_4_ (**Figure 3C**), which was found to be associated with a heightened prevalence of Omicron seropositivity.

### Covaxin induced mild to moderate CD4+T cell responses against WT SARS-CoV-2

**Figure 4:**
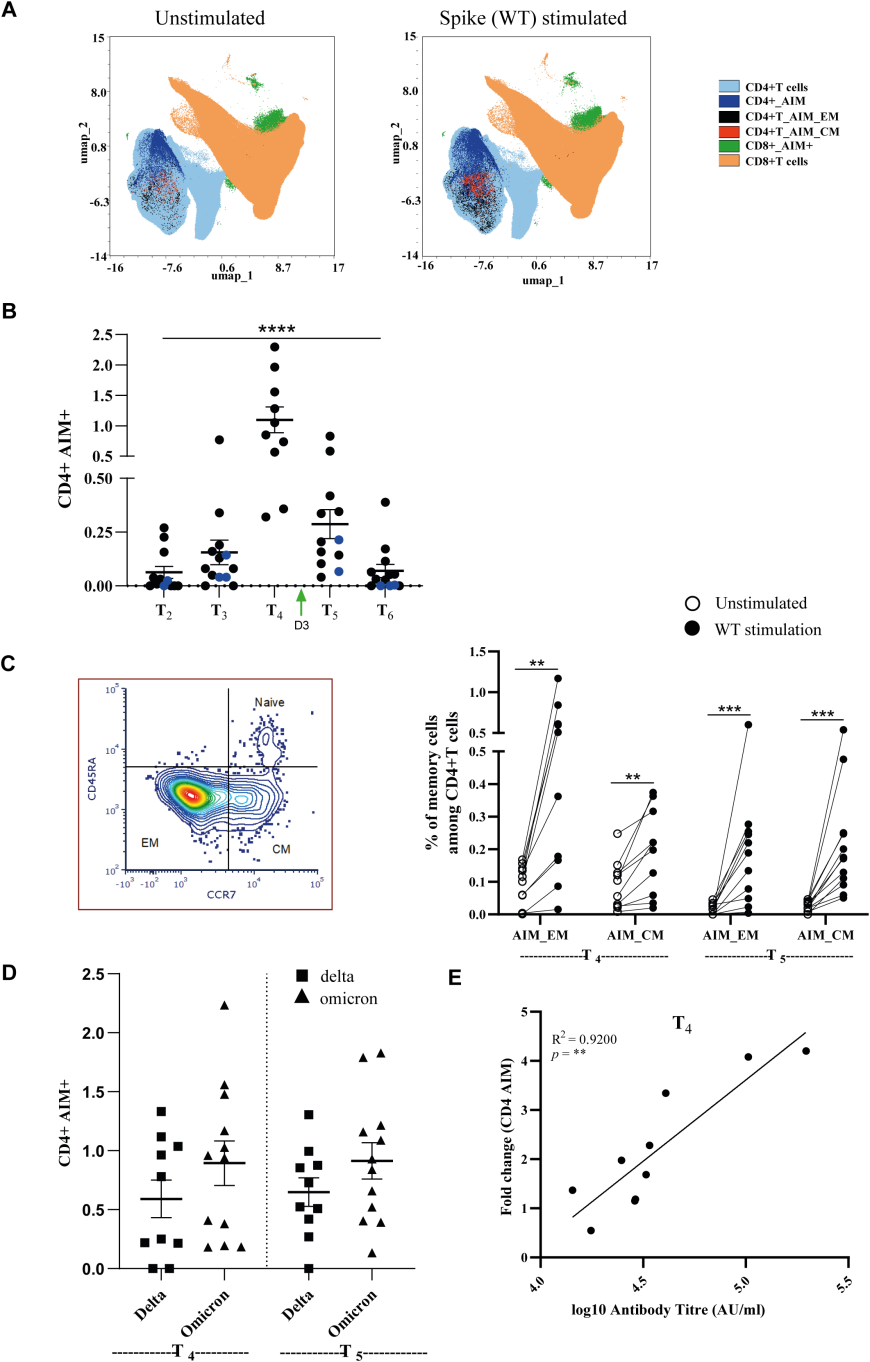
Vaccination induces spike-specific CD4^+^ T-cell responses that are elicited and activated during the Omicron wave and post-third dose of vaccination. **(A)** Unbiased dimensionality reduction by uniform manifold approximation and projection (UMAP) for visualization of CD4 and CD8 cell types (seven parameters) observed from flow cytometric analysis of PBMCs. **(B)** The abundance of spike-specific CD4^+^ T cells compared with the unstimulated group in the longitudinal cohort. The graph represents the percentage of AIM^+^ CD4^+^ T cells after background subtraction of the unstimulated samples. Black: Follow-up candidates (*n* = 10). Blue: Non-follow-up candidates (*n* = 3). **(C)** Percentage of spike-specific memory T-cell subsets during the Omicron breakthrough (T4) and post-third dose of vaccination (T5). The left panel represents the gating criteria for memory cell differentiation. **(D)** CD4^+^ T-cell responses against mutated variants of SARS-CoV-2 spike (S) following before (T4) and after the third dose of vaccination (T5). **(E)** Correlations depicting the fold change in spike-specific CD4^+^ T-cell frequency compared to paired antibody titer at T_4_ time points. Data represented as mean ± SEM. **p < 0.01, ***p < 0.001, ****p < 0.0001. Significant differences were tested by Student’s *t*-test.

To test for the generation of CD4^+^ T-cell responses against WT SARS-CoV-2 following Covaxin administration, we managed to follow 13 donors from the cohort at longitudinal time points. Blood samples were collected at five different time points: T_2_, 3 months; T_3_, 6 months; T_4_, 12 months; T_5_, 18 months after the second dose and 10 days after the third dose; and T_6_, 24 months after the second dose and 6 months after the third dose of vaccination. For this assay, the PBMCs were stimulated with spike synthetic peptides/peptivator pool for 24 h followed by FACS staining for estimation of T-cell activation status. UMAP-based clustering of single-cell data revealed distinct T-cell populations, demonstrating differential abundance between unstimulated and stimulated conditions (**Figure 4A**).

After 24 h of antigen challenge, the percentage of AIM+ (CD154^+^CD137^+^), AIM+EM+ (CD45RA^−^CCR7^−^), and AIM+CM+ (CD45^−^CCR7^+^) T cells was estimated by full-spectra flow cytometry. The results are represented as the AIM response of CD4^+^ T cells in five different time points with 13 participants (**Figure 4B**). Our data revealed that subjects vaccinated with the third dose (T_5_) of the vaccine and after 12 months of the second dose (T_4_) demonstrated enhanced AIM+ CD4 T cells (**Figure 4B**). In contrast, CD8^+^ T cells showed comparatively minimal AIM induction, indicating a predominantly CD4^+^-driven cellular immune response (**Supplementary Figure 4**). Moreover, we found that the percentage of EM and CM cells at T_4_ and T_5_ significantly increased (*p* < 0.01) in stimulated relative to unstimulated T cells (**Figure 4C**).

When we analyzed for cross-reactivity with Delta and Omicron, we observed that there was an increase in the frequency of AIM+ CD4^+^ T cells relative to the unstimulated controls, suggesting that Delta and Omicron variant spike peptides were cross-reactive with WT SARS-CoV-2 and therefore resulted in increased immune protection (**Figure 4D**, **Supplementary Figure 2**).

The percentage of spike-specific CD4^+^ T-cell responses, being consistently higher (**Figure 4E**), was confirmed by a strong correlation between antibody titers at T_4_.

The results above indicate that it was clear that Covaxin induces mild to moderate immune responses in T cells. To estimate the frequency of subjects who were optimally responding to Covaxin, we calculated the SI for CD4^+^ T cells. The magnitude of SARS-CoV-2 reactive T-cell responses was measured using SI taking AIM+ expression into consideration (17). All of the subjects were recruited between January 2021 and January 2023, which included convalescent as well as uninfected vaccinees. The stimulation index for CD4^+^ T cells was calculated with interquartile range (**Supplementary Figure 3**). Samples within the interquartile range and above Q3 were classified as high responders, while those below Q1 were considered low responders. Analysis revealed that only 50% of the 60 individuals mounted an optimal CD4^+^ T-cell response following Covaxin administration.

## Discussion

The current study attempted to bridge a substantial gap regarding the understanding of the longitudinal impact of emerging SARS-CoV-2 infection or inactivated virus-based vaccine administration on humoral and cell-mediated immunity. Here, we concentrated on analyzing antibody and T-cell responses in a cohort of Covaxin-vaccinated subjects as a follow-up study for 2 years. Full-spectra multicolor flow cytometry was used to evaluate the CD4^+^ and CD8^+^ T-cell responses and their subtypes and effector function in response to Covaxin and its cross-reactivity with Delta and Omicron SARS-CoV-2 variants. In addition, we measured the serum spike-specific IgG and neutralizing antibody titers longitudinally in our cohort. To the best of our knowledge, this study reports for the first time on a 2-year longitudinal follow-up profiling of spike RBD antibody and T-cell responses after administration of the inactivated virus-based vaccine Covaxin (BBV152).

According to the findings of our longitudinal serosurvey (**Figure 2A**), IgG antibody titers significantly increased following the administration of the second dose of Covaxin. After the first dose, approximately 40%–45% of subjects were non-responders. Additionally, 15%–20% of Covaxin recipients did not exhibit any response to the vaccination overall. Similar proportions of early non-responders and interindividual variability in antibody responses have also been reported following mRNA (BNT162b2, mRNA-1273) and adenoviral vector vaccines (ChAdOx1 nCoV-19), highlighting a common feature of SARS-CoV-2 vaccine-induced immunity across platforms (18–21). We also observed a gradual decrease in IgG titers after vaccination, which was boosted strongly during the Omicron infection wave of January–March 2022. Infection with the Omicron variant induced a strong antibody response in almost 100% subjects, and the antibody titers were maintained for 1 year. This finding suggested that the rise in seroprevalence was largely due to natural infection with the Omicron variant during India’s third COVID-19 wave (January–March 2022), which reported over 20 million cases (22–25). These results, which were seen in convalescent and uninfected vaccine recipients, were undoubtedly caused by both a natural infection and COVID-19 vaccination (25–27). Following getting a sharp increase in antibody titers at 12 months after the second dose and Omicron infection, our aim was to examine the neutralizing response at all-time intervals, from T_0_ to T_6_ (**Figure 3A**). We found that 99% of study participants had a complete virus-neutralizing response after Omicron infection, but more research is needed to determine whether neutralization titers correlate with receptor-binding domain IgG antibodies throughout all time points.

The fundamental understanding of T-cell responses to SARS-CoV-2 at various stages of immunization is crucial. Here, we used PBMCs collected from vaccinated subjects to provide functional validation of CD4^+^ and CD8^+^ T-cell responses. Spike-specific peptide pools of predominant SARS-CoV-2 variants were included in the investigations. Previous longitudinal studies of mRNA and adenovirus vaccines have consistently shown that T-cell responses are more durable than antibody responses, supporting the importance of evaluating cellular immunity over extended periods (28–30).

Our study demonstrated that CD4^+^ T cells play a predominant role in cross-reactivity against SARS-CoV-2 and the formation of memory responses, with CD8^+^ T cells contributing minimally (**Figure 4**, **Supplementary Figure 4**). This observation aligns with the findings from other cohorts, which also reported limited involvement of CD8^+^ T cells among the cross-reactive T cells (31, 32). Most individuals vaccinated with Covaxin or who recovered from moderate COVID-19 lacked SARS-CoV-2-specific CD8^+^ T-cell responses. This deficiency could be due to poor memory CD8^+^ T-cell formation or functional impairment. Further research is needed to understand the role of CD8^+^ T cells in SARS-CoV-2 pathogenesis across different populations and disease outcomes. In parallel, spike antigen stimulation resulted in an increased frequency of memory B cells, indicating a strong humoral memory response that may contribute to long-term immune protection (**Supplementary Figure 5**).

The antigen-specific T-cell response was predominantly characterized by a Th1 phenotype, with a substantial increase in cytokines such as IL-8 and IFN-γ (**Figure 3C**). This aligns with earlier studies that also reported Th1-dominant cytokine responses and minimal Th2 responses in COVID-19-positive, vaccinated individuals (33–36). Notably, the majority of participants displayed detectable levels of virus-specific CD4^+^ T cells across all five time points, with a significant response observed during the Omicron wave and after the third vaccination (**Figure 4B**). Our study found that spike-specific CD4^+^ T cells (AIM) were significantly elevated at time point 4 (T_4_), which corresponded to the Omicron wave. This increase may be associated with the presence of asymptomatic Omicron infections. Additionally, antibody profile analysis revealed heightened spike-specific responses among vaccinated individuals during the Omicron wave, and both parameters are highly correlated (**Figure 4E**) (37). Longitudinal analyses of mRNA vaccine recipients have demonstrated persistence of spike-specific CD4^+^ T cells for up to 12 months, supporting the durability of cellular immunity observed in our cohort (38).

The observed increase in memory phenotype response at these two time points is consistent with our antibody titer and neutralization results, which demonstrated a more pronounced antigenic response compared to other time points (T_2_, T_3_, T_6_) (36–39).

The collection of samples for this study (**Figure 4**) was performed at a time when the Delta and Omicron VOCs existed, and this allowed us to ascertain how vaccine-induced CD4^+^ T cells react with the spike antigen of the ancestral strain, Delta, and Omicron variants. Our data indicated that the extent of the antigen-specific T-cell response against all variants in our investigation was comparable, despite multiple alterations in the Delta and Omicron variants (40, 41). This is probably due to the presence of T cells with specificity for conserved epitope regions of the ancestral strain, which would likely cross-react with emerging variants (42, 43).

Participants who received the vaccine consistently produced a significant CD4^+^ T-cell response against SARS-CoV-2. Using the stimulation index as the criterion, similar outcomes were reached (**Supplementary Figure 3**). The analysis revealed that only ~50% of the individuals in the total population were responders, which was similar to other published reports (31, 32).

The next generation of vaccines must pay close attention to the extent of T-cell responses induced upon vaccination given the significance of T-cell memory in limiting disease severity and safeguarding against emerging variants. However, few studies (43, 44) have looked into the factors that contribute to strong T-cell immune responses against SARS-CoV-2. When this study was planned, there was limited information on vaccine-induced T-cell immunity against SARS-CoV-2. Therefore, the main goal of the study was to track T-cell responses against the virus over an extended period post-vaccination.

The COVID-19 incidence burden on the Indian subcontinent is significant, despite the fact that case fatality rates were low, which may be attributed to the presence of highly cross-reactive CD4^+^ T cells. Understanding the role of cross-reactive CD4^+^ T cells in disease outcome and immunological memory formation is crucial for future COVID-19 vaccine development and implementation.

### Limitations of the study

Limitations of our study include the fact that, although the cohort size was relatively large, participants were drawn from a single geographic and demographic population, which may limit the generalizability of the findings to other groups with different genetic, environmental, or epidemiological backgrounds. Additionally, as this was an observational study, it does not allow definitive conclusions about the causal protective effect of Covaxin against SARS-CoV-2 infection or disease severity. Finally, the lack of a direct comparative analysis with Covishield, a widely used COVID-19 vaccine in India, limits the interpretation of relative immunogenicity and effectiveness across vaccine platforms.

## Data Availability

The datasets presented in this study can be found online, using the URL: https://doi.org/10.1101/2023.06.02.23290825.
