# Supplementary material for: Long-term humoral and cellular immune responses following Covaxin vaccination: a 2-year prospective longitudinal study
Source: Front Immunol. 2026 Mar 4;17:1754692. doi: 10.3389/fimmu.2026.1754692 (PMC13010997; doi:10.3389/fimmu.2026.1754692)
Supplement: Supplementary file 1 [file DataSheet1.pdf]

Supplementary Figure 1

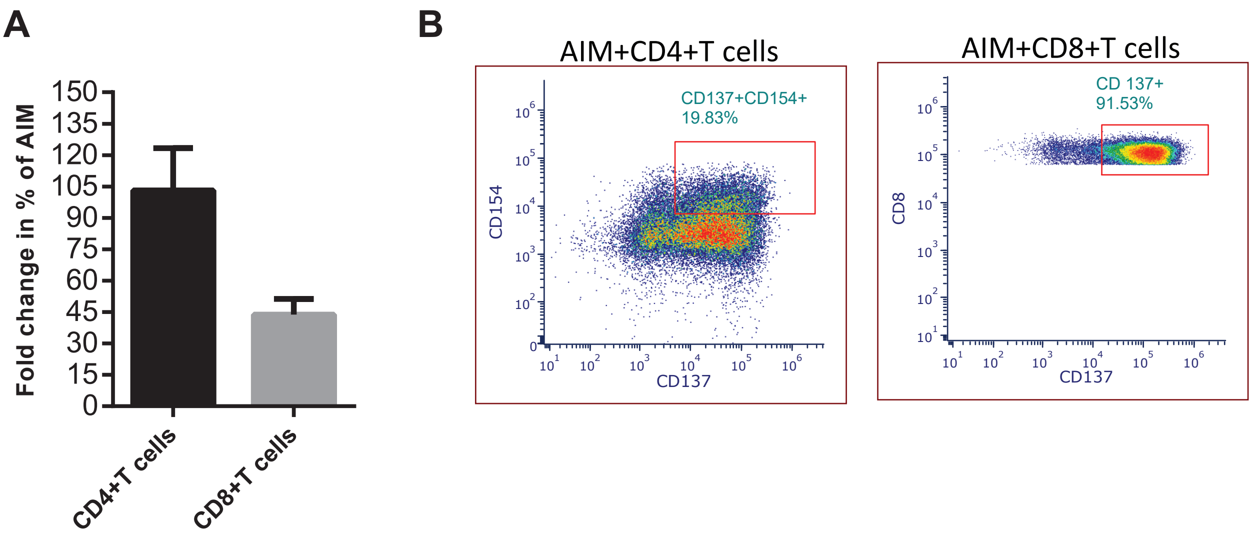

Supplementary Figure 1: Enhanced AIM+ CD4+ and CD8+T Cell Responses Upon Cytostim Stimulation

Bar plot represents the increase in fold change in % if AIM+CD4+ and CD8+T cells after stimulation with Cytostim (Figure A). Representative flow cytometry plots showing the gating of AIM+CD4+ and AIM+CD8+ T cells (Figure B). Bars represent mean ± SEM.

Supplementary Figure 2

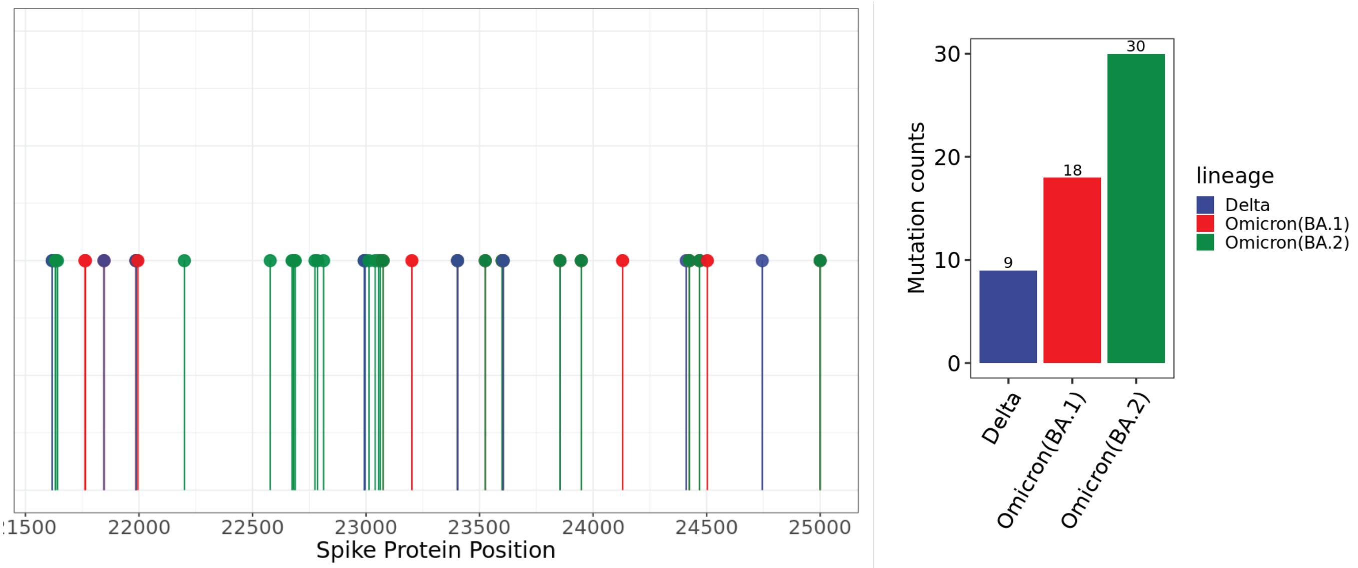

Supplementary Figure 2 : Mutation load distribution and mutation frequency in VoCs of SARS-CoV-2.

Supplementary Figure 3

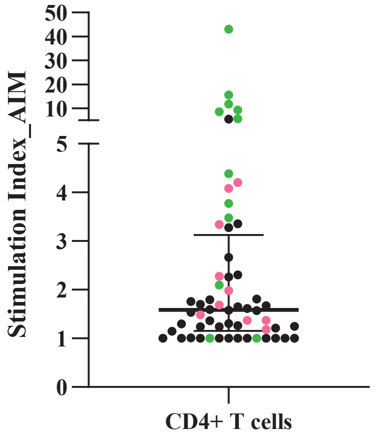

Supplementary Figure 3: Scatter Plot of Spike-Specific CD4⁺ T Cell Stimulation Index with Median and IQR

Scatter dot-plot with median and interquartile range (IQR) showing the distribution of spike specific stimulation index for CD4+T cells. Pink and Green circles represent patients at T4 and T5 time point respectively (n=60).

Supplementary Figure 4

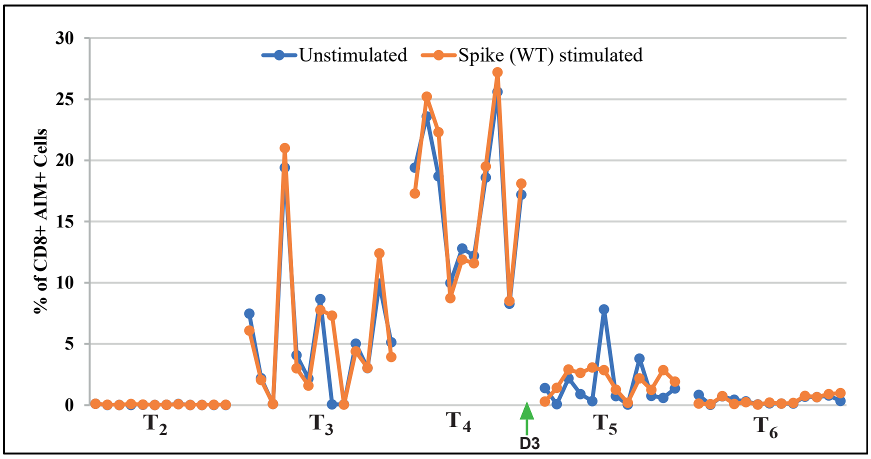

Supplementary Figure 4: Longitudinal Analysis of CD8⁺ T Cell Activation in Response to Spike Antigen

Statistics comparing the abundance of spike-specific CD8+T cells in between unstimulated and stimulated group for the longitudinal cohort

Supplementary Figure 5

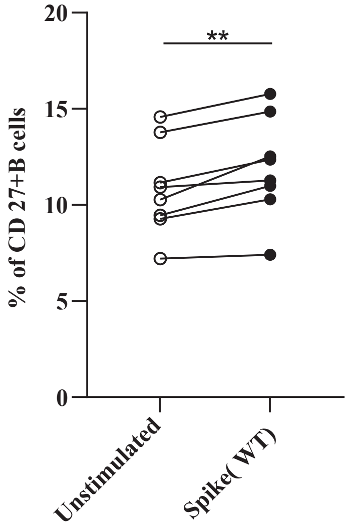

Supplementary Figure 5: Memory B Cell Response at T5 in Unstimulated vs. Stimulated Groups

At the T5 time point, the stimulated group exhibited an increased percentage of memory B cells compared to the unstimulated group. Bars represent mean + SEM. **p<0.01. Significant differences were tested by paired Student's t-test.
